# Challenges in surveillance and response

**DOI:** 10.1186/1475-2875-11-S1-O3

**Published:** 2012-10-15

**Authors:** Laurence Slutsker

**Affiliations:** 1 Center for Global Health CDC, Atlanta, USA

## 

Surveillance in the context of malaria elimination will needs to shift from measuring reductions in morbidity and mortality to detecting infections (with or without symptoms). The malaria elimination surveillance research and development agenda needs to develop tools and strategies for active and prompt detection of infection. The capacity to assess trends and respond without delay will need to be developed, so that surveillance itself becomes an intervention. Research is needed to develop sensitive field tests that can detect low levels of parasitaemia and/or evidence of recent infection. Examples of recent work on surveillance and response issues in several African countries will be discussed to illustrate approaches in active case detection and case investigations, cell phone reporting and response, and strategies to access mobile populations.

